# Giving Pure Shift NMR Spectroscopy a REST—Ultrahigh-Resolution Mixture Analysis

**DOI:** 10.1021/acs.analchem.2c02411

**Published:** 2022-09-07

**Authors:** Marshall J. Smith, Laura Castañar, Ralph W. Adams, Gareth A. Morris, Mathias Nilsson

**Affiliations:** Department of Chemistry, University of Manchester, Oxford Road, Manchester M13 9PL, U.K.

## Abstract

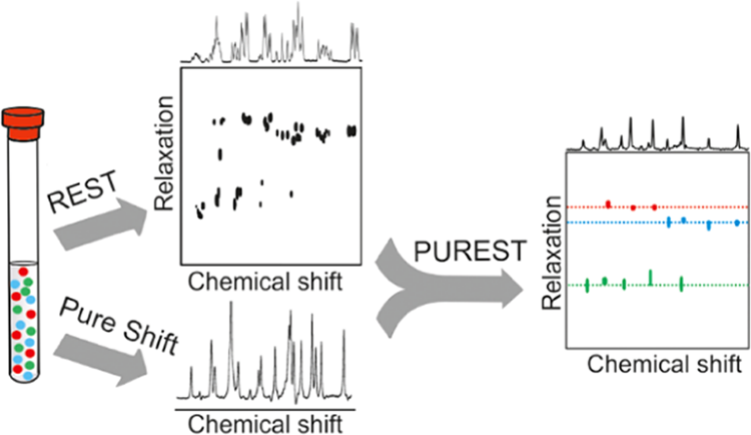

Most interesting problems in chemistry, biology, and pharmacy involve mixtures. However, analysis of such mixtures by NMR remains a challenge, often requiring the mixture components to be physically separated before analysis. A variety of methods have been proposed that exploit species-specific properties such as diffusion and relaxation to distinguish between the signals of different components in a mixture without the need for laborious separation. However, these methods can struggle to distinguish between components when signals overlap. Here, we exploit the relaxation properties of selected nuclei to distinguish between different components of a mixture while using pure shift methods to increase spectral resolution by up to an order of magnitude, greatly reducing signal overlap. The advantages of the new method are demonstrated in a mixture of d-xylose and l-arabinose, distinguishing unambiguously between the five major species present.

NMR spectroscopy offers unparalleled insights into the structures and dynamics of molecules. However, analysis of mixtures, especially in one-dimensional (1D) NMR, can be challenging. In ^1^H spectra, the narrow chemical shift range and prominent signal multiplicity often cause signals to overlap, complicating analysis. Two-dimensional (2D) techniques such as COSY,^1^ HSQC,^2^ and TOCSY^3^ can alleviate some of the spectral overlaps, making structural information more accessible but still struggle in complicated spin systems and mixtures. For example, determining the number of species present in a mixture can be laborious at best and often impossible using these methods. Here, a new method is described that exploits relaxation labeling of spin systems to distinguish the signals of individual species in mixtures.

One powerful technique that can be used to determine the number of species present in a mixture is diffusion NMR, where differences in self-diffusion rates between species are exploited to distinguish between their signals.^4^ This method is useful in mixtures where there are significant differences in diffusion coefficient between components and where signals do not overlap in the NMR spectrum. However, diffusion experiments are of limited value in cases where diffusion coefficients are similar (e.g., for isomers).

In 2017, a new experiment, relaxation-encoded selective TOCSY (REST),^5^ was proposed that sought to tackle this limitation of diffusion NMR experiments by exploiting relaxation instead of diffusion. Each spin in a molecule experiences different local magnetic fields, produced by interactions with surrounding nuclei and electrons, causing different relaxation rates. Each signal in a spectrum, therefore, will, in general, have a different relaxation rate. Using the same logic as diffusion-ordered spectroscopy (DOSY),^6^ a relaxation-ordered (ROSY)^7^ plot can be constructed in which signal amplitude is plotted as a function of chemical shift and relaxation time or rate constant.^7^ Such a spectrum would normally not identify different signals that belong to the same molecule since they will, in general, all have different relaxation times. However, in REST experiments, the relaxation weighting of selected coherences is propagated throughout entire spin systems so that each signal within a given spin system acquires the same relaxation weighting. Generating a ROSY plot then allows signals that are part of the same spin system to be identified, as their peaks align at the same relaxation time/rate, just as the peaks align at the same diffusion coefficient in a DOSY spectrum.

Relaxation weighting can be achieved using methods such as inversion recovery (IR)^8^ for longitudinal *T*_1_ relaxation weighting and periodic refocusing of *J* evolution by coherence transfer (PROJECT),^9^ for transverse *T*_2_ relaxation. Fitting signal recovery (IR) or attenuation (PROJECT) to an exponential allows the relaxation time constant to be determined for each signal. Where there is little signal overlap, REST experiments can prove very useful for distinguishing the signals of species that have similar diffusion coefficients but differences in relaxation.^10^ Here, the more difficult case where there is substantial signal overlap in the conventional ^1^H spectrum is attacked.

The analysis of relaxation or diffusion data usually requires the assumption that each signal in a spectrum originates from a single species, and hence that signal evolution in a relaxation or diffusion experiment can be fitted to a monoexponential function (of time or gradient amplitude squared, respectively). Where signals with different relaxation/diffusion overlap, signal evolution will be multiexponential, and fitting with a single exponential will give a compromise result.^10^ Fitting to a biexponential function requires excellent data quality and is only practical where the decay constants differ substantially.^10,11^ Other powerful post-acquisition methods such as component resolution (CORE),^12^ speedy component resolution (SCORE),^13^ and optimized unmixing of true spectra for component resolution (OUTSCORE)^14^ extract component spectra and decay constants from diffusion/relaxation data by exploiting the fact that all non-exchanging signals from a given species show the same diffusion coefficient or relaxation constant. However, even these methods struggle in cases of severe signal overlap, large differences in component concentration, or similar diffusion coefficient/decay constant.

One of the best ways to avoid multiexponential signal decays is, unsurprisingly, to avoid signal overlap by increasing the resolution of the NMR spectrum. One way of increasing resolution is to use a stronger magnetic field, but this can be very expensive and currently has a physical limit of 28.2 Tesla (1.2 GHz ^1^H frequency) for commercial instrumentation.^15^ A more accessible approach is to use pure shift NMR methods, which reduce spectral complexity by suppressing the effects of homonuclear scalar coupling (*J*_HH_).^16^ Ideally, each chemical shift is then represented by a well-resolved singlet. Such broadband homonuclear decoupling is typically achieved by generating a time-domain signal in which *J*_HH_ evolution is periodically refocused at a rate that is fast compared to *J*_HH_, either in real time or parameter-time. Pulse sequence elements that refocus homonuclear couplings include the Zangger-Sterk (ZS),^17^ pure shift yielded by chirp excitation (PSYCHE),^18^ and bilinear rotation decoupling (BIRD)^19^ methods. Real-time (RT) methods^20^ periodically interrupt the measured FID with a suitable *J*-refocusing element. Parameter-time methods^17^ construct a time-domain interferogram using a pseudo-2D experiment with a *J*-refocusing element at the midpoint of an evolution time *t*_1_.

In the new method described here, the PSYCHE element is used in parameter-time acquisition experiments to achieve broadband homonuclear decoupling in REST. The pure shift relaxation-encoded selective TOCSY (PUREST) delivers a step-function improvement in the resolution of the REST experiment, allowing for the analysis of very challenging mixtures such as those of carbohydrates.

## Results and Discussion

**Figure 1:**
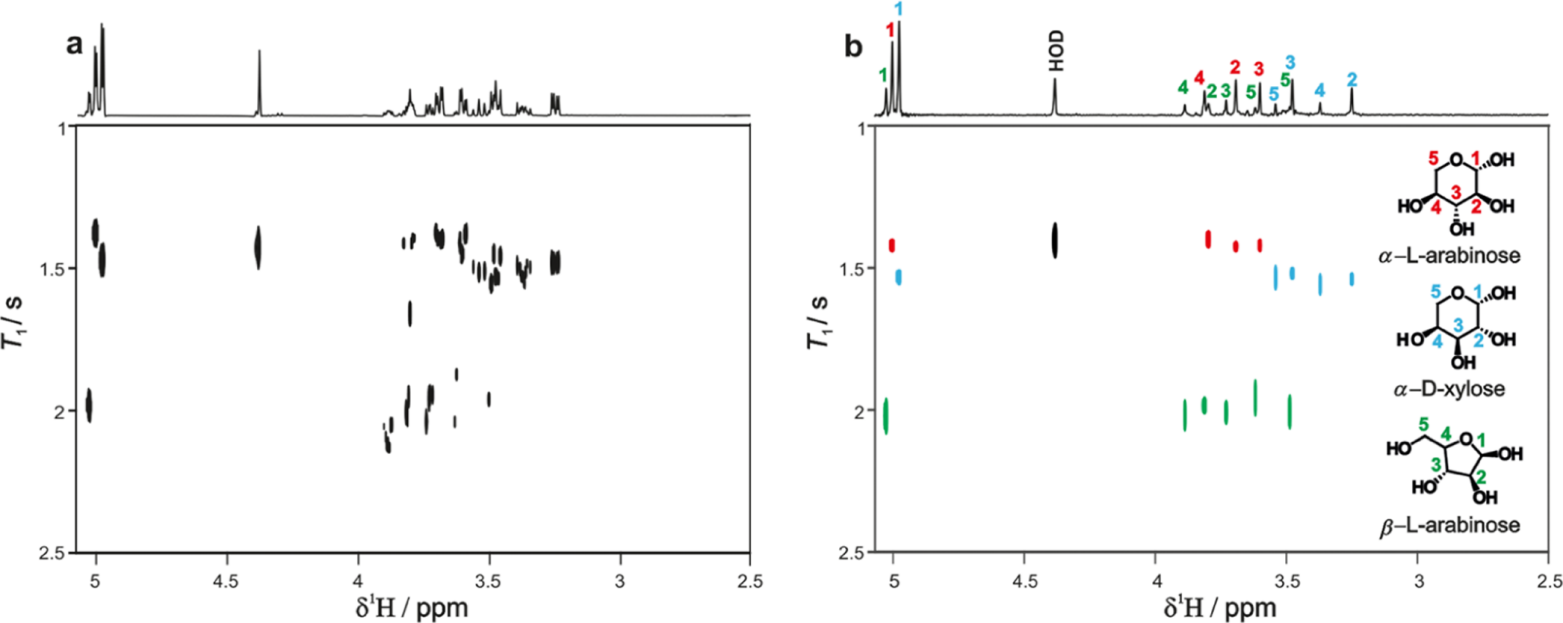
REST-*T*_1_ (left) and PUREST-*T*_1_ (right) spectra for a solution of arabinose and xylose. The top spectra correspond to the first increment in REST and PUREST, respectively. The pulse sequence of Figure 3 was used, with IR relaxation weighting. The relaxation delay τ varied from 0.001 to 12 s in 16 steps. Full experimental parameters are provided in the Supporting Information (SI).

Xylose and arabinose are attractive sources for bio-based fuel and chemical production and frequently occur as mixtures.^21^ However, there is substantial signal overlap in the ^1^H NMR spectrum of a mixture of d-xylose and l-arabinose (Figure S3). In the following, PUREST experiments are used to untwine the signals of the five species present in this mixture. Figure 1 compares the results of the previous REST-*T*_1_ and the new PUREST-*T*_1_ experiments, using IR for the relaxation weighting. The region around 5 ppm was selected for relaxation encoding, and monoexponential fitting was used (the relaxation times are too similar to be distinguished with biexponential fitting, which could be an alternative for overlapping signals in more favorable cases). This region contains the anomeric signals of the pyranose forms of α-l-arabinose and α-d-xylose and the furanose form of β-l-arabinose. In an ideal REST experiment, a ROSY plot would allow the signals of the three spin systems to be clearly distinguished, with three distinct rows of cross-peaks seen. However, in the region 3.2–3.8 ppm, there is substantial spectral overlap, and REST (Figure 1a) struggles to separate the signals. In the PUREST spectrum (Figure 1b), in contrast, the almost complete elimination of signal overlap allows essentially perfect separation of the signals of the three species. (The protons at position 5 in α-l-arabinose are not observed in either experiment because the small *J*_H4H5_ limits the effectiveness of the TOCSY transfer.)^22^

**Figure 2:**
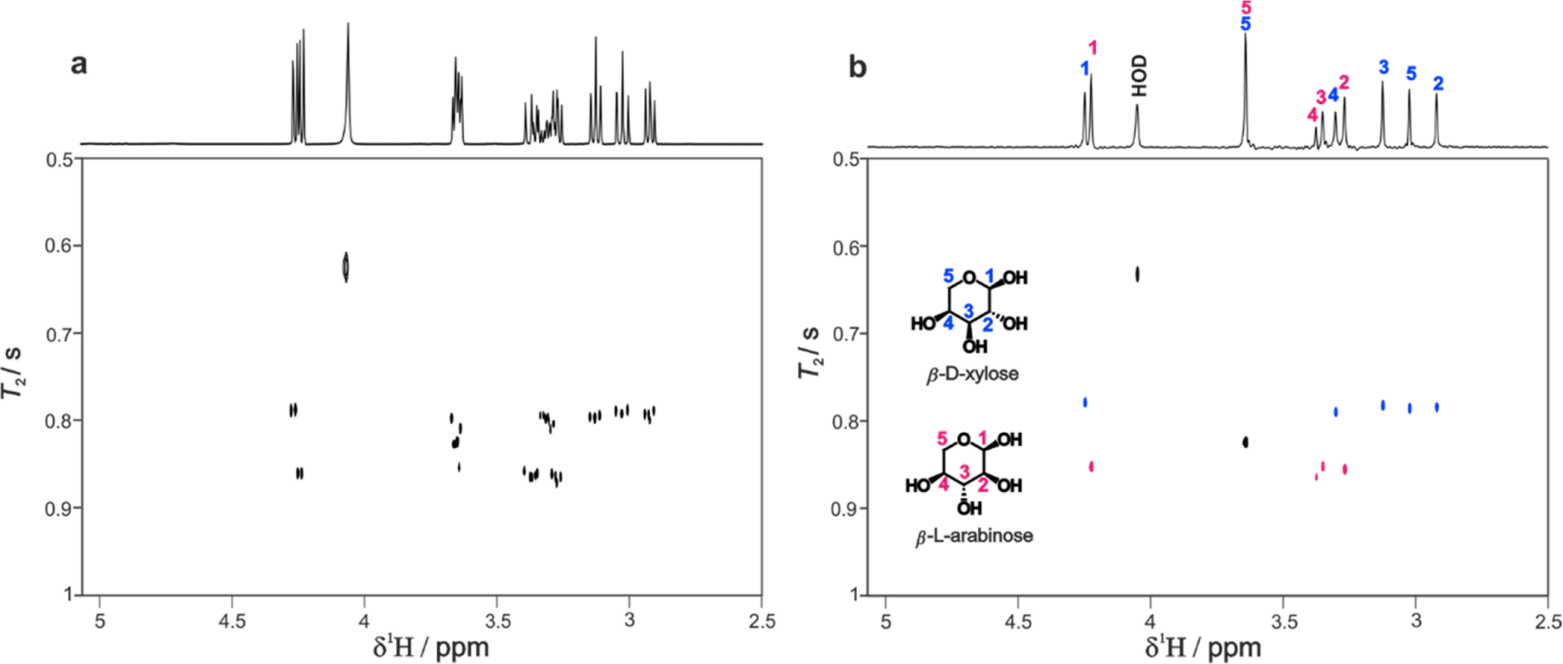
Comparison of REST-*T*_2_ (left) and PUREST-*T*_2_ (right) spectra measured for the arabinose/xylose mixture using the pulse sequence of Figure 3. PROJECT relaxation *T*_2_ weighting was used with 16 increments in the relaxation domain, with loop count (*n*) varying from 10 to 120 and a delay τ of 3 ms. Full experimental parameters are provided in the SI.

Figure 2 compares the results of the previous REST-*T*_2_ (Figure 2a) and the new PUREST-*T*_2_ (Figure 2b) experiments, using PROJECT^9^ relaxation weighting in each case. REST-*T*_2_ allows most of the signals of the pyranose forms β-l-arabinose and β-d-xylose to be distinguished, but the increase in the resolution in PUREST allows H3 and H4 (3.4 ppm) of β-l-arabinose, which are overlapped in the REST spectrum, to be resolved. In the case of H5, however, the near-exact chemical shift degeneracy between β-l-arabinose and β-d-xylose means that an intermediate value of *T*_2_ relaxation time is observed in both REST and PUREST spectra.

## Experimental Section

**Figure 3:**
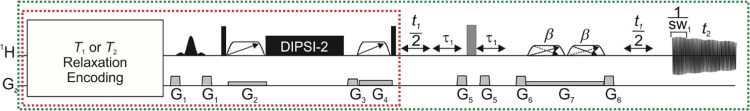
Pulse sequences for REST (red box) and PUREST (green box) using IR or PROJECT relaxation weighting and a PSYCHE *J*-refocusing element. The narrow black rectangles represent 90° hard pulses, and the gray rectangles represent 180° hard pulses. The shaped black pulse represents a 180° frequency-selective refocusing pulse. The trapezoids with a single arrow represent chirp ZQS pulses, and the trapezoids with double arrows represent low flip angle (β) saltire chirp pulses. The light gray trapezoids indicate field gradients; G_3_ is a homospoil gradient, and G_1_, G_5_, and G_6_ are used to enforce the coherence transfer pathway. The wide gray rectangles indicate long, weak rectangular gradient pulses. The truncated FID indicates interferogram acquisition, in which a short chunk of data (duration 1/SW_1_) is acquired for each *t*_1_ increment. The delay τ_1_ is set to 1/4SW_1_. Further information is provided in the SI.

The pulse sequences used to obtain the spectra in Figures 1 and 2 are essentially modular, as shown in Figure 3. The first part of the sequence only, boxed in red, is used for the REST, and the full sequence is used for the corresponding PUREST experiment. Each sequence begins with relaxation weighting, i.e., inversion recovery (IR)^8^ for REST-*T*_1_ and PROJECT^9^ for REST-*T*_2_. Alternative weighting elements can be used, such as saturation recovery for *T*_1_ or transverse relaxation unmodulated echo (TRUE)^23^ for *T*_2_. Relaxation weighting is followed by a selective TOCSY^24^ element that combines a 180° selective refocusing pulse, a zero quantum suppression (ZQS) element,^25^ and DIPSI-2^26^ isotropic mixing to propagate the relaxation-encoded coherence of the selected spins across their respective spin systems. The selective pulse can be used to select a single spectral region with multiple spin systems, or to select multiple regions (see Figure S4 in the SI). As the signals for a given spin system all originate from the magnetization of a single site (selected by the 180° selective pulse), the same relaxation weighting is seen for all signals of a given spin system. The IR relaxation weighting (Figure S1) uses an incremented τ delay; the PROJECT *T*_2_ weighting in Figure S2 uses a fixed τ delay and a variable loop count *n*. The echo time 4τ is short in comparison to 1/*J*_HH_ to minimize homonuclear *J*-modulation but long enough that sample heating is minimal. Although relaxation weighting is used purely qualitatively in the examples shown, it is possible to use it quantitatively if the recovery delay is sufficient. It should be noted, however, that the presence of rapid convection could lead to faster apparent relaxation rates as molecules move into and out of the active volume. A simple and effective way to slow the convection greatly is to use a smaller inside diameter NMR tube.^27,28^ The PSYCHE *J*-refocusing element was used in the PUREST experiments as it generally gives good sensitivity and spectral purity. As PSYCHE is only viable in interferogram acquisition, this was used here rather than real-time. The cost in acquisition time was outweighed in this case by the substantial improvement in the resolution. The greater speed of RT acquisition is attractive but would require the use of a different pure shift element such as ZS or BIRD. These elements were unsuitable in this case as ZS would cause severe line broadening due to the long selective pulse durations required, and BIRD would lead to a large sensitivity penalty.^16^ In any pure shift experiment, a sample-dependent compromise is necessary between sensitivity and spectral purity, and strong coupling will inevitably degrade the latter to some extent.

All NMR spectra were recorded on a Bruker Avance NEO 500 spectrometer with a 5 mm TBI or BBO probe, with nominal maximum *z*-gradient strengths of 67 and 50 G cm^–1^, respectively. The sample of d-xylose and l-arabinose was 500 mM in each component, in DMSO-*d*_6_: D_2_O 4:1 v/v, with TSP added as a reference. *T*_1_ experiments were conducted at 280 K; experiment times for REST-*T*_1_ and PUREST-*T*_1_ were 1 h 12 min and 8 h 57 min, respectively. *T*_2_ experiments were conducted at 303 K; experiment times for REST-*T*_2_ and PUREST-*T*_2_ were 1 h 8 min and 11 h 40 min, respectively. All isotropic mixing elements used DIPSI-2^18^ with a duration of 0.2 s. The spectral window in all cases was set to 5000 Hz (10 ppm). All ROSY spectra were produced using the general NMR analysis toolbox (GNAT).^29^ Further description of the experimental procedure is provided in the SI.

## Conclusions

An ultrahigh-resolution form of the REST experiment, PUREST, using pure shift methods to remove the effects of homonuclear scalar coupling, has been developed and demonstrated. The method successfully disentangles the proton NMR signals of all five major species present in a mixture of xylose and arabinose. The increase in resolution in the frequency domain obtained almost completely eliminates the ambiguity in the relaxation domain caused by signal overlap. The price paid for the very high resolution obtained is a somewhat longer acquisition time; in applications with greater chemical shift differences between coupled partners (allowing for short selective pulses to be used, reducing *T*_2_ losses), this could be avoided using Zangger-Sterk real-time pure shift data acquisition. These experiments come with a significant cost in sensitivity and may not be practical with low concentration samples. Naturally, sensitivity would be greatly enhanced using a high field instrument with a cryogenic probe. Alternatives to pure shift REST include very selective 1D TOCSY,^30^ e.g., using GEMSTONE,^31,32^ and *F*_1_-pure shift TOCSY with very high *F*_1_ digitization.^33^

## Supporting Information Available

Further experimental results, guidance, and pulse sequence code for Bruker spectrometers (PDF)

## Notes

All experimental data, pulse sequence codes, wave files, and macros can be downloaded from DOI: 10.48420/20004170.

## References

[ref1] Aue W. P.; Bartholdi E.; Ernst R. R. Two-dimensional spectroscopy. Application to nuclear magnetic resonance. J. Chem. Phys. 1976, 64, 2229–2246. 10.1063/1.432450.

[ref2] Bodenhausen G.; Ruben D. J. Natural abundance nitrogen-15 NMR by enhanced heteronuclear spectroscopy. Chem. Phys. Lett. 1980, 69, 185–189. 10.1016/0009-2614(80)80041-8.

[ref3] Braunschweiler L.; Ernst R. R. Coherence transfer by isotropic mixing: Application to proton correlation spectroscopy. J. Magn. Reson. 1983, 53, 521–528. 10.1016/0022-2364(83)90226-3.

[ref4] Morris G. A. Diffusion-Ordered Spectroscopy. eMagRes, 2007.

[ref5] Dal Poggetto G.; Castanar L.; Adams R. W.; Morris G. A.; Nilsson M. Relaxation-encoded NMR experiments for mixture analysis: REST and beer. Chem. Commun. 2017, 53, 7461–7464. 10.1039/C7CC03150E. 28567463

[ref6] Morris K. F.; Johnson C. S. Diffusion-ordered two-dimensional nuclear magnetic resonance spectroscopy. J. Am. Chem. Soc. 1992, 114, 3139–3141. 10.1021/ja00034a071.

[ref7] Nishiyama Y.; Frey M. H.; Mukasa S.; Utsumi H. 13C solid-state NMR chromatography by magic angle spinning 1H T1 relaxation ordered spectroscopy. J. Magn. Reson. 2010, 202, 135–139. 10.1016/j.jmr.2009.10.009. 19900827

[ref8] Drain L. E. A Direct Method of Measuring Nuclear Spin-Lattice Relaxation Times. Proc. Phys. Soc. Sect. A 1949, 62, 301–306. 10.1088/0370-1298/62/5/306.

[ref9] Aguilar J. A.; Nilsson M.; Bodenhausen G.; Morris G. A. Spin echo NMR spectra without J modulation. Chem. Commun. 2012, 48, 811–813. 10.1039/C1CC16699A. 22143456

[ref10] Nilsson M.; Connell M. A.; Davis A. L.; Morris G. A. Biexponential Fitting of Diffusion-Ordered NMR Data: Practicalities and Limitations. Anal. Chem. 2006, 78, 3040–3045. 10.1021/ac060034a. 16642991

[ref11] Istratov A. A.; Vyvenko O. F. Exponential analysis in physical phenomena. Rev. Sci. Instrum. 1999, 70, 1233–1257. 10.1063/1.1149581.

[ref12] Stilbs P.; Paulsen K.; Griffiths P. C. Global Least-Squares Analysis of Large, Correlated Spectral Data Sets: Application to Component-Resolved FT-PGSE NMR Spectroscopy. J. Phys. Chem. A 1996, 100, 8180–8189. 10.1021/jp9535607.

[ref13] Nilsson M.; Morris G. A. Speedy component resolution: An improved tool for processing diffusion-ordered spectroscopy data. Anal. Chem. 2008, 80, 3777–3782. 10.1021/ac7025833. 18407669

[ref14] Colbourne A. A.; Meier S.; Morris G. A.; Nilsson M. Unmixing the NMR spectra of similar species – vive la différence. Chem. Commun. 2013, 49, 10510–10512. 10.1039/c3cc46228e. 24088897

[ref15] Schwalbe H. Editorial: New 1.2 GHz NMR Spectrometers- New Horizons?. Angew. Chem., Int. Ed. 2017, 56, 10252–10253. 10.1002/anie.201705936. 28738448

[ref16] Zangger K. Pure shift NMR. Prog. Nucl. Magn. Reson. Spectrosc. 2015, 86-87, 1–20. 10.1016/j.pnmrs.2015.02.002. 25919196

[ref17] Zangger K.; Sterk H. Homonuclear Broadband-Decoupled NMR Spectra. J. Magn. Reson. 1997, 124, 486–489. 10.1006/jmre.1996.1063.

[ref18] Foroozandeh M.; Adams R. W.; Meharry N. J.; Jeannerat D.; Nilsson M.; Morris G. A. Ultrahigh-Resolution NMR Spectroscopy. Angew. Chem., Int. Ed. 2014, 53, 6990–6992. 10.1002/anie.201404111. PMC4320760 24861024

[ref19] Garbow J. R.; Weitekamp D. P.; Pines A. Bilinear Rotation Decoupling of Homonuclear Scalar Interactions. Chem. Phys. Lett. 1982, 93, 504–509. 10.1016/0009-2614(82)83229-6.

[ref20] Lupulescu A.; Olsen G. L.; Frydman L. Toward single-shot pure-shift solution 1H NMR by trains of BIRD-based homonuclear decoupling. J. Magn. Reson. 2012, 218, 141–146. 10.1016/j.jmr.2012.02.018. 22446507

[ref21] Verhoeven M. D.; De Valk S. C.; Daran J.-M. G.; Van Maris A. J. A.; Pronk J. T. Fermentation of glucose-xylose-arabinose mixtures by a synthetic consortium of single-sugar-fermenting Saccharomyces cerevisiae strains. FEMS Yeast Res. 2018, 18 (8), 1–12. 10.1093/femsyr/foy075. 30010916

[ref22] Bubb W. A. NMR spectroscopy in the study of carbohydrates: Characterizing the structural complexity. Concepts Magn. Reson. 2003, 19A, 1–19. 10.1002/cmr.a.10080.

[ref23] Kiraly P.; Dal Poggetto G.; Castañar L.; Nilsson M.; Deák A.; Morris G. A. Broadband measurement of true transverse relaxation rates in systems with coupled protons: application to the study of conformational exchange. Chem. Sci. 2021, 12, 11538–11547. 10.1039/D1SC03391C. 34667556 PMC8447259

[ref24] Kessler H.; Oschkinat H.; Griesinger C.; Bermel W. Transformation of homonuclear two-dimensional NMR techniques into one-dimensional techniques using Gaussian pulses. J. Magn. Reson. 1986, 70, 106–133. 10.1016/0022-2364(86)90366-5.

[ref25] Thrippleton M. J.; Keeler J. Elimination of Zero-Quantum Interference in Two-Dimensional NMR Spectra. Angew. Chem., Int. Ed. 2003, 42, 3938–3941. 10.1002/anie.200351947. 12949874

[ref26] Rucker S. P.; Shaka A. J. Broadband homonuclear cross polarization in 2D N.M.R. using DIPSI-2. Mol. Phys. 1989, 68, 509–517. 10.1080/00268978900102331.

[ref27] Barbosa T. M.; Rittner R.; Tormena C. F.; Morris G. A.; Nilsson M. Convection in liquid-state NMR: expect the unexpected. RSC Adv. 2016, 6, 95173–95176. 10.1039/C6RA23427E.

[ref28] Swan I.; Reid M.; Howe P. W. A.; Connell M. A.; Nilsson M.; Moore M. A.; Morris G. A. Sample convection in liquid-state NMR: Why it is always with us, and what we can do about it. J. Magn. Reson. 2015, 252, 120–129. 10.1016/j.jmr.2014.12.006. 25681799

[ref29] Castañar L.; Poggetto G. D.; Colbourne A. A.; Morris G. A.; Nilsson M. The GNAT: A new tool for processing NMR data. Magn. Reson. Chem. 2018, 56, 546–558. 10.1002/mrc.4717. 29396867 PMC6001793

[ref30] Dal Poggetto G.; Castanar L.; Morris G. A.; Nilsson M. A new tool for NMR analysis of complex systems: selective pure shift TOCSY. RSC Adv. 2016, 6, 100063–100066. 10.1039/C6RA22807K.

[ref31] Kiraly P.; Nilsson M.; Morris G. A.; Adams R. W. Single-scan ultra-selective 1D total correlation spectroscopy. Chem. Comm. 2021, 57, 2368–2371. 10.1039/D0CC08033K. 33533774

[ref32] Kiraly P.; Kern N.; Plesniak M. P.; Nilsson M.; Procter D. J.; Morris G. A.; Adams R. W. Single-Scan Selective Excitation of Individual NMR Signals in Overlapping Multiplets. Angew. Chem., Int. Ed. 2021, 60, 666–669. 10.1002/anie.202011642. PMC7839668 32965750

[ref33] Foroozandeh M.; Adams R. W.; Nilsson M.; Morris G. A. Ultrahigh-Resolution Total Correlation NMR Spectroscopy. J. Am. Chem. Soc. 2014, 136, 11867–11869. 10.1021/ja507201t. 25111063

