# Supplementary material for: Giving Pure Shift NMR Spectroscopy a REST—Ultrahigh-Resolution Mixture Analysis
Source: Anal Chem. 2022 Sep 7;94(37):12757–61. doi: 10.1021/acs.analchem.2c02411 (PMC9494296; doi:10.1021/acs.analchem.2c02411)
Supplement: Supplementary file 1 — ac2c02411_si_001.pdf [file ac2c02411_si_001.pdf]

Giving Pure Shift NMR Spectroscopy a REST– Ultrahigh Resolution Mixture Analysis

Supporting Information

*Marshall J. Smith, Laura Castañar, Ralph W. Adams, Gareth A. Morris, and Mathias Nilsson**

Mathias.Nilsson@manchester.ac.uk

Table of Contents

A. Scheme of work 3

B. Experimental section 3

1. Details of pulse sequences 3
2. Data processing using the General NMR Analysis Toolbox (GNAT) 5
   - 2.1. Reconstructing homonuclear decoupled interferograms in GNAT 5
   - 2.2. Processing relaxation data 5

C. Experimental data 6

1. Sample preparation and ^{1}H NMR spectrum 6
2. PUREST-T_1 using phase modulated pulses to select multiple frequencies 7

D. Pulse sequences in Bruker format 8

1. PUREST-T_1 - Inversion Recovery (IR) with CW presaturation and option between ZS and PSYCHE 8
2. PUREST-T_1 - with full wavemaker compatibility, multiple frequency selective pulse and option between ZS and PSYCHE 14
3. PUREST-T_2 – using PSYCHE 20

A. Scheme of work

The following section describes a suggested workflow for obtaining optimal data using the PUREST family of experiments. Steps that are not strictly necessary are marked as optional.

1) Identify the overlapped signals of interest for which coupling correlations are sought. Optimise the 180° selective shaped pulse (typically RSNOB or REBURP) to select only these signals.
2) Optimise the DIPSI-2 isotropic mixing to transfer magnetization from the selected signals to the rest of their respective spin systems, using a selective 1D TOCSY experiment. (optional)
3) Measure their T_1 and T_2 relaxation times, e.g. using inversion recovery (IR) and periodic refocussing of J evolution by coherence transfer (PROJECT) respectively.
4) Select the relaxation weighting method that gives the greatest difference in relaxation time between the spins of interest.
5) Acquire a pure shift spectrum using either the Zangger-Sterk (ZS) or the Pure Shift Yielded by Chirp Excitation (PSYCHE) active spin refocusing (ASR) element. Optimise the chunk duration, number of chunks and ASR element parameters (bandwidth of the 180° selective pulse and gradient amplitude for ZS; flip angle β, sweep width, duration and gradient amplitude for PSYCHE).
6) Run the PUREST experiment with the optimised selective pulse, isotropic mixing and pure shift parameters.
7) Process the raw 3D data using the Bruker AU macro "pshift" or the General NMR Analysis Toolbox (GNAT); both are freely available at http://nmr.chemistry.manchester.ac.uk.
8) Process and analyse the reconstructed pseudo 2D relaxation data, e.g. using the GNAT.

B. Experimental section

All data were acquired on a 500 MHz Bruker Avance Neo spectrometer, running Topspin 4.0.7. T_2 data were acquired with a 5 mm TBI probe equipped with a z-gradient coil with a maximum nominal gradient strength of 0.67 T m^{-1}. T_1 data were acquired with a 5 mm BBFO probe equipped with a z-gradient coil with a maximum nominal gradient strength of 0.50 T m^{-1}. (The change of probe was necessary as the TBI developed a fault during the investigation).

1. Details of pulse sequences

Detailed pulse sequences (Figures S1 and S2), along with the appropriate phase cycling (Tables 1 and 2 respectively), are shown below. White rectangles represent spherical randomisation pulses (duration 1-2 ms). Black narrow rectangles represent hard 90° pulses and grey narrow rectangles represent hard 180° pulses. The shaped, wide, black pulse represents a 180° selective pulse, typically RSNOB or REBURP. DIPSI-2 isotropic mixing is used with a duration of 50-200 ms depending on the spin system to be investigated. The white trapezoids with a single arrow on either side of the DIPSI-2 element represent low power 180° chirp pulses for zero quantum coherence suppression. In this work all zero quantum suppression pulses used a 20 kHz bandwidth with the first pulse having a 10 ms duration and the second pulse having a 30 ms duration. The PSYCHE element used two low flip angle (β) frequency-swept saltire pulses, represented by white trapezoids with two arrows. Typically, 10 kHz sweep range, 30 ms duration and a 20° flip angle gave adequate results. Gradient

pulses G_2, G_6 and G_7 had 1 ms duration and amplitudes of 15.4, 31.5 and 40.81 G cm^{-1}, respectively. These gradients are used to enforce the coherence transfer pathway. Gradient pulses G_3 and G_5 are weak gradient pulses (amplitudes 2.01 and 2.68 G cm^{-1}) applied simultaneously with the chirp pulses to suppress zero quantum coherences. G_1 and G_4 are homospoil gradient pulses with amplitudes of 24.8 and 20.8 G cm^{-1} and duration 1 - 2 ms. G_8 is a weak field gradient (amplitude 2 G cm^{-1}) applied during the saltire pulses to suppress unwanted coherence transfer pathways. All gradient pulses were followed by a 1 ms stabilisation delay. The discontinuous FID represents the interferogram acquisition mode used in the PUREST experiments, to yield pseudo-3D homonuclear decoupled datasets. Short chunks of data ($1/SW_1$) are acquired (one for each t_1 increment) to minimise J evolution during the chunks. Typically the duration of each chunk is 10 – 20 ms. J was refocused in the middle of the data chunk by setting τ_1 to $1/4SW_1$. The number of chunks to be acquired depends on the spin system under investigation and the desired resolution of the spectrum. In this work, 16 chunks were acquired for PUREST-T_1 and 40 chunks for PUREST-T_2 data. A 5 s recovery delay, d1, was used throughout.

The T_1 experiments were performed at 280 K. The inversion recovery delay τ (Figure S1) is an incremented delay defined in a variable delay (VD) list that should contain at least 8 values to allow adequate exponential fitting. In the work presented, 16 increments were acquired in the relaxation domain, with a VD list from 0.001 to 12 s.

The T_2 data were acquired at 303 K. The interpulse delay τ (Figure S2) in the PROJECT element should be << $1/J_{HH}$ but long enough to minimise the effects of sample heating; this work used a 3 ms τ delay. The total echo time is defined by the total duration of the perfect echo element multiplied by the number of cycles, n, defined in the user-defined VC list, with values of 6 – 100 used in this work. A minimum of 8 different n values is required for good exponential fitting; in this work 16 increments were used in the relaxation domain.

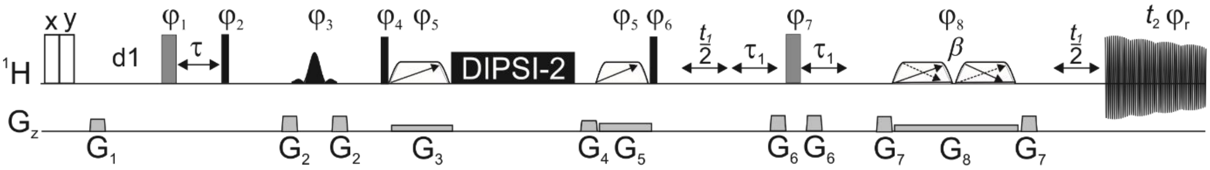

Figure S1: Pulse sequence of a PUREST experiment using inversion recovery relaxation weighting (PUREST-T_1) and PSYCHE as the ASR element.

TABLE S1: Phase cycling for the PUREST-T_1 experiment.

φ_1	x_4, $-x_4$
φ_2	x_8, $-x_8$
φ_3	x, y, -x, -y
φ_6	x_{16}, y_{16}
$\varphi_{4,5,7,8}$	x
φ_r	$(x\ -x)_4$, $(-x\ x)_4$, $(y\ -y)_4$, $(-y\ y)_4$

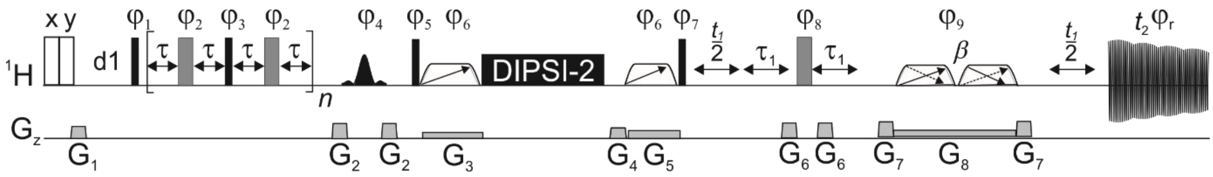

Figure S2: Pulse sequence of a PUREST experiment using PROJECT relaxation weighting (PUREST-T_2) and PSYCHE as the ASR element.

TABLE S2: Phase cycling for the PUREST-T_2 experiment.

$\varphi_{1,2,5,6,7,8}$	x
φ_3	y
φ_4	x, y, -x, -y
φ_9	x_4, y_4, $-x_4$, $-y_4$
φ_r	$(x\ -x)_2$, $(-x\ x)_2$

2. Data processing instructions using the General NMR Analysis Toolbox (GNAT)

The pseudo 3D, broadband homonuclear decoupled, raw datasets were processed using the General NMR Analysis Toolbox, GNAT, (freely available at http://nmr.chemistry.manchester.ac.uk) to reconstruct the 2D interferograms. All relaxation data were processed in the MATLAB version of GNAT (version 1.1.3), which is compatible with MATLAB 2019 or higher. All relaxation data used peak heights for the analysis unless otherwise stated.

2.1 Reconstructing homonuclear decoupled interferograms in GNAT

Import the raw pseudo 3D or pseudo 2D data into the GNAT by navigating to Files (top left) and selecting the manufacturer of the spectrometer used to acquire the data. Under the "Pure shift" tab either manually enter the interferogram acquisition parameters (chunk duration, number of points per chunk and number of dropped points), or use the "Estim. Param." function to identify the parameters automatically. Finally, use the "convert" function to construct the homonuclear decoupled interferograms. Standard processing including, but not limited to, Fourier transformation, baseline correction, phase correction and apodization can then be performed.

2.2 Processing relaxation data

Once the pseudo 2D data have been imported and processed (section 2.1), the relaxation data may be analysed. Firstly, ensure that the values of the variable delays or numbers of cycles have been imported correctly, by navigating to Edit > Settings and choosing the relaxation tab in the pop-up window. Complete echo durations in CPMG and PROJECT are calculated from the loop counters by using the "Convert" function in this window. Once the delays are correct, the settings window may be closed. To do the fitting for the relaxation data, navigate to the "Relaxation" tab on the right-hand side. Using the ROSY module, the user can determine the fitting routine to be performed, fitting either longitudinal, T_1, or transverse, T_2, relaxation data. The longitudinal relaxation processing uses a 3 parameter fit, transverse relaxation processing uses a 2 parameter fit. In addition, the user can determine whether the fitting routine uses peak picking or integrals. To use peak picking a threshold is required to be set in either the first (for T_2 data, corresponding to the smallest loop count) or the last (for T_1 data, corresponding to longest variable delay) spectrum. To

use integrals, the first or last spectrum should be chosen as for the peak picking routine. The integral regions can then be set under the “Analysis” tab in the “Integrate” module. Selecting “Run” in the ROSY module will generate a 2D ROSY plot of relaxation against frequency in a new window. Individual spectra in an array can be excluded from fitting if necessary by using the “Prune” module at the left hand side of the main processing window.

C. Experimental data

1. Sample preparation and ^{1}H NMR spectrum

52 mg of commercial D-xylose (Sigma-Aldrich) and L-arabinose (Fluorochem) were dissolved in a mixture of 0.7 µL DMSO-d_6:D_2O in an 4:1 *v*/*v* mixture, and a small amount of TSP was added. Figure S3 shows the ^{1}H spectrum of this mixture.

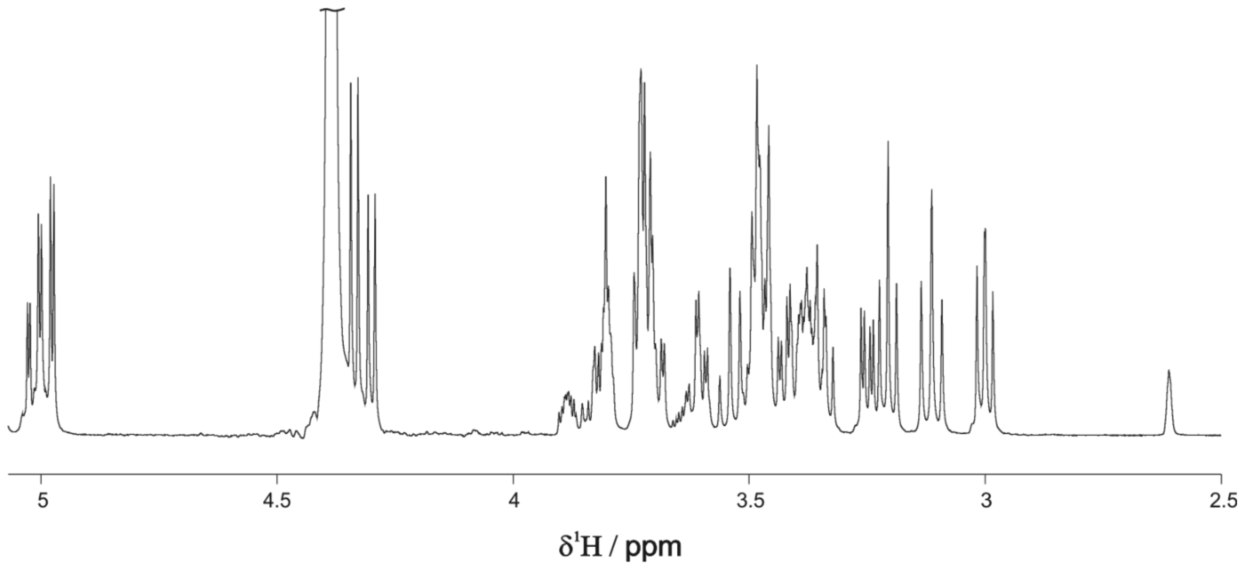

Figure S3. ^{1}H NMR spectrum for a mixture of D-xylose and L-arabinose dissolved in 4:1 v/*v* mixture of DMSO-d_6:D_2O.

2. Selecting multiple signals with phase-modulated pulses in PUREST experiments

Phase-modulated pulses can be used in PUREST experiments to select multiple signal regions that have spectral overlap in a single experiment. Figure S4 shows, as a proof of principle, the results from a PUREST-T_1 experiment that was frequency selective for regions around both 4.9 and 4.3 ppm. The selective pulse in this case was generated using the Wavemaker functionality in the Bruker Topspin software.

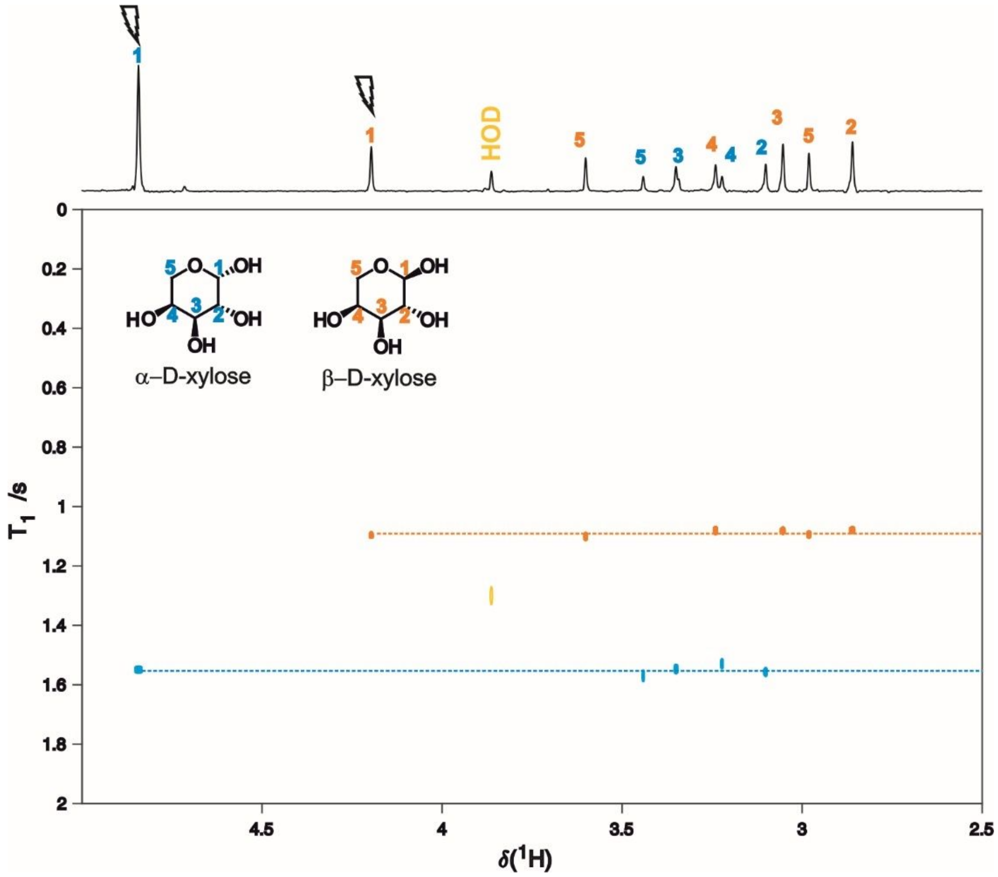

Figure S4: PUREST-T_1 experiment on a sample of 500 mM D-xylose in DMSO-d_6:D_2O (4:1) *v/v*. Inversion recovery was used for the relaxation encoding with 8 increments in the relaxation domain with linear steps from 0.001 s to 25 s. Zangger-Sterk homonuclear decoupling was implemented using a 30 Hz bandwidth RSNOB pulse with a simultaneous z-encoding gradient of 0.2 G cm^{-1}. The initial selective pulse of the selective TOCSY element was a phase modulated pulse generated by Wavemaker selecting for two regions, 4.9 and 4.3 ppm, each with a 40 Hz bandwidth. A recovery delay of 7 s was used before each transient.

D. Pulse sequences in Bruker format

1. PUREST-T_1 - Inversion Recovery (IR) with CW presaturation and option between ZS and PSYCHE (for pure shift T_1 measurements)

```
;purest_t1_irprcwvd
;
; Pure shift T1-relaxation encoded selective TOCSY (PUREST-T1)
;           measurement using inversion recovery and
;           saturation pulses prior to d1
;           continuous wave presaturation is applied during d1 and VD
;           DIPSI-2 with ZQF.
;           Option between ZS and PSYCHE pure shift ASR elements
;
; University of Manchester
; Manchester NMR methodology group
; https://www.nmr.chemistry.manchester.ac.uk/
;
; Mathias Nilsson
; Mathias.Nilsson@manchester.ac.uk
;
; Marshall Smith
; marshall.smith@manchester.ac.uk
;
; Avance II+/III Version
; Topspin 4.x
;
;$CLASS=HighRes
;$DIM=3D
;$TYPE=
;$SUBTYPE=
;$COMMENT=
;
;Pure shift psudo 3D experiment
;    with interferogram acquisition
;      ZGOPTION= -DZS (Zangger-Sterk on)
;      ZGOPTION= -DdSALTIRE (PSYCHE on)

#include <Avance.incl>
#include <Delay.incl>
#include <Grad.incl>

define delay tauA
define delay tauB

;PULSES
"p2=p1*2"
"p3=p1"
"p18=1m"
"p19=1m"
"p11=p40"

; Selective TOCSY
"FACTOR1=(d9/(p6*115.112))/2"
"l1=FACTOR1*2"

;DELAYS
```

```
"in0=inf1/2"
"d0=0"
"tauA=in0/2-p18-d18-50u"
"tauB=dw*2*cnst4"
"d18=1m"
"d19=1m"
"d11=30m"
"d11=30m+1s/(cnst12)-1s/(cnst12)"
"d11=30m"

;OFFSETS
"spoff12=bf1*(cnst22/1000000)-o1"
"spoff22=0"
"spoff32=0"
"spoff34=0"

;Zero Quantum filter calculation
"p33=1000000.0/(cnst53*4)"
"cnst33= (p33/p1) * (p33/p1)"
"spw32=plw1/cnst33"
"p35=1000000.0/(cnst54*4)"
"cnst35= (p35/p1) * (p35/p1)"
"spw34=plw1/cnst35"

#   ifdef dSALTIRE
;PSYCHE element
;Double Saltire pulse calculations
"cnst50=(cnst20/360)*sqrt((2*cnst21)/(p40/2000000))"
"p30=1000000.0/(cnst50*4)"
"cnst31= (p30/p1) * (p30/p1)"
"spw40=plw1/cnst31"
"spoff40=0"
#   endif

;Acquistion
"acqt0=0"
baseopt_echo

1 ze
2 d11 pl1:f1

  10u UNBLKGRAD
  p8 ph21
  p8 ph22
  p18:gp8*-1
  d18 BLKGRAD

50u LOCKH_OFF
1m fq=cnst29(bf ppm):f1
d12 pl9:f1
d1 cw:f1 ph29
4u do:f1
1m fq=cnst30:f1
d12 pl1:f1
50u LOCKH_ON
50u UNBLKGRAMP

  p2 ph1
```

```
4u fq=cnst29(bf ppm):f1
4u pl9:f1
vd cw:f1 ph29
4u do:f1
4u fq=cnst30:f1
4u pl1:f1

  p1 ph2
  p16:gp1
  d16 pl0:f1
  p12:sp12:f1 ph3                              ;selective 180r
  p16:gp1
  d16 pl1:f1

3 p1 ph4
  20u gron0
  (p32:sp32 ph5):f1
  10u groff
  500u pl10:f1

                                                ;begin DIPSI2
4 p6*3.556 ph23
  p6*4.556 ph25
  p6*3.222 ph23
  p6*3.167 ph25
  p6*0.333 ph23
  p6*2.722 ph25
  p6*4.167 ph23
  p6*2.944 ph25
  p6*4.111 ph23

  p6*3.556 ph25
  p6*4.556 ph23
  p6*3.222 ph25
  p6*3.167 ph23
  p6*0.333 ph25
  p6*2.722 ph23
  p6*4.167 ph25
  p6*2.944 ph23
  p6*4.111 ph25

  p6*3.556 ph25
  p6*4.556 ph23
  p6*3.222 ph25
  p6*3.167 ph23
  p6*0.333 ph25
  p6*2.722 ph23
  p6*4.167 ph25
  p6*2.944 ph23
  p6*4.111 ph25

  p6*3.556 ph23
  p6*4.556 ph25
  p6*3.222 ph23
  p6*3.167 ph25
  p6*0.333 ph23
  p6*2.722 ph25
  p6*4.167 ph23
  p6*2.944 ph25
```

```
 p6*4.111 ph23
 lo to 4 times l1
                                                  ;end DIPSI2
5  p17:gp2
 d17 pl0:f1
 10u gron10
 (p34:sp34 ph5):f1               ;Zero Quantum Filter
 20u groff
 20u pl1:f1

 p1 ph6

6  d0
        tauA        ;spin echo
 50u
 p18:gp3
 d18
 p2 ph7
 p18:gp3
 d18
 50u
 tauA

        tauB                                                       ; Delay for drop points

 50u          ;selective spin echo
 d19 pl0:f1
 p19:gp4
 d19
# ifdef ZS
 10u gron20                                    ;spatial encoding
 (p22:sp22 ph8):f1
 10u groff                                     ;spatial encoding
# endif

# ifdef dSALTIRE
        10u
 ( center (p40:sp40 ph8):f1 (p11:gp11) )
        10u
# endif

        d19
 p19:gp4
 d19
 50u BLKGRAMP

 d0

 go=2 ph31
 d11 mc #0 to 2
 F1QF(id0)
 F2QF(ivd)
exit
50u LOCKH_OFF

ph1=0 0 0 0 2 2 2 2
ph2=0 0 0 0 0 0 0 0 2 2 2 2 2 2 2 2
ph3=0 1 2 3
```

```
ph4=0
ph5=0
ph6=0 0 0 0 0 0 0 0 0 0 0 0 0 0 0 0 1 1 1 1 1 1 1 1 1 1 1 1 1 1 1 1
ph7=0
ph8=0
ph21=0
ph22=1
ph23=3
ph25=1
ph29=0
ph31=0 2 0 2 0 2 0 2 2 0 2 0 2 0 2 0 1 3 1 3 1 3 1 3 3 1 3 1 3 1 3 1


;POWER LEVEL
;pl0  : zero power (0W)
;pl1  : power level for pulse (default)
;pl10 : power level for TOCSY-spinlock
;spw12 : power level of refocusing shaped pulse
;spw22 : power level for ZS refocusing pulse
;spw32 : power level of adiabatic pulse of first ZQF element
;spw34 : power level of adiabatic pulse of last ZQF element
;spw40 : power level of double-chirp PSYCHE pulse element

;PULSE DURATION
;p1  : 90 degree high power pulse
;p2  : 180 degree high power pulse
;p6  : 90 degree low power pulse
;p8  : saturation recovery pulse                                      [1-1.5 ms]
;p12 : 180 degree refocusing shaped pulse
;     choose p12 according to desired selectivity
;p22 : 180 degree refocusing ASR shaped pulse
;p32 : first ZQF 180 degree inversion shaped pulse (adiabatic)         [10 ms]
;p34 : second ZQF 180 degree inversion shaped pulse (adiabatic)        [30 ms]
;p40 : duration of PYSCHE pulse

;GRADIENT DURATION
;p11 : duration of gradient under PSYCHE pulse
;p16 : duration of CTP gradients for selective pulse                   [1 ms]
;p17 : duration of CTP gradients for z-TOCSY                           [1 ms]
;p18 : saturation recovery gradient pulse                              [1 ms]
;p19 : duration of CTP gradients for ZS selective pulse [1 msec]

;DELAY
;d1  : relaxation delay;                                               [2-10 s]
;d9  : TOCSY mixing time                                               [50-200 ms]
;d11 : delay for disk I/O                                              [30 ms]
;d16 : recovery delay for gradients of selective pulse CTP             [1 ms]
;d17 : selective spin-echo delay in selective-TOCSY block              [200 us]
;d18 : recovery delay for saturation gradients
                          [1ms]
;d19 : recovery delay for CTP gradients for ZS and PSYCHE selective pulse [1 msec]

;PULSE SHAPE
;spnam12 : file name for the selective 180 refocusing shaped pulse for selective TOCSY    [RSNOB or REBURP]
;spnam22 : file name for the selective 180 refocusing shaped pulse PS                     [RSNOB or REBURP]
;spnam32 : file name for the adiabatic shaped pulse using in first ZQF                    [CHIRP]
;        smoothed chirp (low to high, 20% smoothing, 1000 points, 20KHz)
;spnam34 : file name for the adiabatic shaped pulse using in last ZQF                     [CHIRP]
```

```
;	smoothed chirp (low to high, 20% smoothing, 1000 points, 20KHz)
;spnam40 : file name for the PSYCHE pulse [SALTIRE]

;GRADIENT SHAPE
;gpnam1 : SMSQ10.100
;gpnam2 : SMSQ10.100
;gpnam3 : SMSQ10.100
;gpnam4 : SMSQ10.100
;gpnam8 : SMSQ10.100
;gpnam11 : RECT.1

;GRADIENT STRENGTH
;gpz0 : first ZQF gradient                         [3%]
;gpz1 : CTP gradient                               [13 or 23%]
;gpz2 : homospoil gradient                         [37%]
;gpz3: CTP gradient                                [49%]
;gpz4: CTP gradient                                [61%]
;gpz8 : saturation recovery gradient               [31%]
;gpz10: last ZQF gradient                          [4%]
;gpz11: PSYCHE gradient                            [1-3%]
;gpz20: ZS z-encoding gradient                     [0-3%]

;CONSTANTS
;cnst4: Number or drop points [4]
;cnst12: chemical shift for first frequency of selective pulse (offset, in ppm)
;cnst20: flip angle for PSYCHE pulse [10-25]
;cnst21: Bandwidth of psyche pulse [10000]
;cnst22: chemical shift for selective pulse (offset, in ppm)
;cnst29: chemical shift for solvent suppression
;cnst41: Bandwidth for selective pulse (Hz)
;cnst53: GammaB1 of first adiabatic ZQF shaped pulse
;cnst54: GammaB1 of last adiabatic ZQF shaped pulse


;OTHER
;td1 : number of delays in VDLIST
;vd : variable delay, taken from vd-list
;define VDLIST
;this pulse program produces a ser-file (PARMOD = 3D)
;in0 : 1/(2 * SW) = DW
;nd0 : 2
;NS: 16 * n, total number of scans: NS * TD0
;DS: 16
```

2. PUREST-T_1 - Inversion Recovery (IR) with option between ZS and PSYCHE and full wavemaker compatibility (for pure shift T_1 measurements). Multiple frequency selective pulse in selective TOCSY

```
;purest_t1_irmf_wvm
;
; Pure shift T1-relaxation encoded selective TOCSY (PUREST-T1)
; measurement using inversion recovery
; All shape pulses wavemaker compatible
; multiple frequency selectivity
; DIPSI-2 with ZQF. Option between ZS and PSYCHE Pure shift
;
; University of Manchester
; Manchester NMR methodology group
; https://www.nmr.chemistry.manchester.ac.uk/
;
; Mathias Nilsson
; Mathias.Nilsson@manchester.ac.uk
;
; Marshall Smith
; marshall.smith@manchester.ac.uk

;University of Manchester
; Marshall Smith
; marshall.smith@manchester.ac.uk
;
;
;Avance II+/III Version
;Topspin 4.x
;
;$CLASS=HighRes
;$DIM=3D
;$TYPE=
;$SUBTYPE=
;$COMMENT=

;Pure shift 3D broad-band experiment
;    with interferogram acquisition
;      ZGOPTION= -DZS (Zangger-Sterk)
;      ZGOPTION= -DdSALTIRE (PSYCHE on)
;
; "wvm -a" command to generate pulse shapes with wavemaker

#include <Avance.incl>
#include <Delay.incl>
#include <Grad.incl>

define delay tauA
define delay tauB

;PULSES
"p2=p1*2"
"p3=p1"
"p18=1m"
```

```
"p19=1m"
;"p11=p50"

; Selective TOCSY
"FACTOR1=(d9/(p6*115.112))/2"
"l1=FACTOR1*2"

;DELAYS
"in0=inf1/2"
"d0=0"
"tauA=in0/2-p18-d18-50u"
"tauB=dw*2*cnst4"
"d18=1m"
"d19=1m"
"d11=30m"
"d11=30m+1s/(cnst12)-1s/(cnst12)"
"d11=30m+1s/(cnst13)-1s/(cnst13)"
"d11=30m+1s/(cnst41)-1s/(cnst41)"
"d11=30m"

;OFFSETS

"spoff22=0"
"spoff32=0"
"spoff34=0"
"cnst30=0"

#   ifdef ZS
"d11=30m+1s/(cnst42)-1s/(cnst42)"
# endif

#   ifdef dSALTIRE
"d11=30m+1s/(1+cnst48)"
"d11=30m+1s/(1+cnst50)"
"d11=30m+1s/(1+cnst51)"
"p50=cnst51*1000.0"
"spw50=plw1/(((1000000.0/(((cnst48/360)*sqrt((2*cnst50*1000)/(p50/1000000)))*4))/p1)*((1000000.0/(((cnst48/36
0)*sqrt((2*cnst50*1000)/(p50/1000000)))*4))/p1))"
#   endif

;Acquistion
"acqt0=0"

baseopt_echo
1 ze
2 d11 pl1:f1

  10u UNBLKGRAMP
  p8 ph21
  p8 ph22
  p18:gp8*-1
  d18 BLKGRAMP

50u BLKGRAD
```

```
50u LOCKH_OFF
d1 pl1:f1
50u LOCKH_ON
50u UNBLKGRAD

; T1 inversion recovery
  p2 ph1
 vd
  p1 ph2

; Selective TOCSY
  p16:gp1
  d16 pl0:f1
  p12:sp12:f1 ph3                        ;selective 180r
  p16:gp1
  d16 pl1:f1

3 p1 ph4
  20u gron0
  (p32:sp32 ph5):f1
  20u groff
  200u pl10:f1

                                          ;begin DIPSI2
4 p6*3.556 ph23
  p6*4.556 ph25
  p6*3.222 ph23
  p6*3.167 ph25
  p6*0.333 ph23
  p6*2.722 ph25
  p6*4.167 ph23
  p6*2.944 ph25
  p6*4.111 ph23

  p6*3.556 ph25
  p6*4.556 ph23
  p6*3.222 ph25
  p6*3.167 ph23
  p6*0.333 ph25
  p6*2.722 ph23
  p6*4.167 ph25
  p6*2.944 ph23
  p6*4.111 ph25

  p6*3.556 ph25
  p6*4.556 ph23
  p6*3.222 ph25
  p6*3.167 ph23
  p6*0.333 ph25
  p6*2.722 ph23
  p6*4.167 ph25
  p6*2.944 ph23
  p6*4.111 ph25
```

```
  p6*3.556 ph23
  p6*4.556 ph25
  p6*3.222 ph23
  p6*3.167 ph25
  p6*0.333 ph23
  p6*2.722 ph25
  p6*4.167 ph23
  p6*2.944 ph25
  p6*4.111 ph23
  lo to 4 times l1
                                              ;end DIPSI2
5  p17:gp2
  d17 pl0:f1
  100u gron10
  (p34:sp34 ph5):f1                ;Zero Quantum Filter
  100u groff
  d17 pl1:f1


 p1 ph6
; Pure shift J refocussing
6  d0
tauA
50u
  p18:gp3
  d18
  p2 ph7
  p18:gp3
  d18
  50u
  tauA

        tauB                                           ; Delay for drop points

  50u
  d19 pl0:f1
  p19:gp4
  d19
# ifdef ZS
10u gron20                                ;spatial encoding
 (p22:sp22 ph8):f1
 10u groff                                ;spatial encoding
# endif

# ifdef dSALTIRE
        10u
 ( center (p50:sp50 ph8):f1 (p11:gp11) )
        10u
# endif

        d19
  p19:gp4
  d19
  50u BLKGRAMP
```

```
  d0

 go=2 ph31
 d11 mc #0 to 2
  F1QF(id0)
  F2QF(ivd)
exit
50u LOCKH_OFF

ph1= 0 0 0 0 2 2 2 2
ph2= 0 0 0 0 0 0 0 0 2 2 2 2 2 2 2 2
ph3= 0 1 2 3
ph4= 0
ph6= 0 0 0 0 0 0 0 0 0 0 0 0 0 0 0 0 1 1 1 1 1 1 1 1 1 1 1 1 1 1 1 1
ph5= 0
ph7=0
ph8=0
ph9=0
ph21=0
ph22=1
ph23=3
ph25=1
ph29=0
ph31= 0 2 0 2 0 2 0 2 2 0 2 0 2 0 2 0 1 3 1 3 1 3 1 3 3 1 3 1 3 1 3 1


;POWER LEVEL
;pl0  : zero power (0W)
;pl1  : power level for pulse (default)
;pl10 : power level for TOCSY-spinlock
;spw12 : power level of refocusing shaped pulse
;spw22 : power level of ZS ASR pulse
;spw32 : power level of adiabatic pulse of first ZQF element
;spw34 : power level of adiabatic pulse of last ZQF element
;spw50: calculated power level of the PSYCHE pulse [35-60 dB]

;PULSE DURATION
;p1  : 90 degree high power pulse
;p2  : 180 degree high power pulse
;p6  : 90 degree low power pulse
;p8  : Saturation recovery pulse                                        [1-1.5 msec]
;p12 : 180 degree refocusing shaped pulse
;     choose p12 according to desired selectivity
;p22 : 180 degree refocusing shaped pulse in ZS element
;p32 : first ZQF 180 degree inversion shaped pulse (adiabatic)          [10 msec]
;p34 : second ZQF 180 degree inversion shaped pulse (adiabatic)         [30 msec]
;p50 : Length of SALTIRE pulse [25-70 ms]

;GRADIENT DURATION
;p11 : duration of gradient pulse under SALTIRE pulse                   [=p50]
;p16 : duration of CTP gradients for Selective pulse                    [1 msec]
;p17 : duration of CTP gradients for z-TOCSY                            [1 msec]
;p18 : saturation recovery gradient pulse                               [1 msec]
```

;p19 : duration of CTP gradients for ZS selective pulse [1 msec]

;DELAY
;d1 : relaxation delay; [2-10 s]
;d9 : TOCSY mixing time [50-200 ms]
;d11 : delay for disk I/O [30 ms]
;d16 : recovery delay for gradients of selective pulse CTP [1 ms]
;d17 : selective spin-echo delay in selective-TOCSY block [200 us]
;d18 : recovery delay for saturation gradients [1ms]
;d19 : recovery delay for CTP gradients for ZS selective pulse [1 ms]

;PULSE SHAPE
;spnam12 : file name for the selective 180 refocusing shaped pulse for selective TOCSY [RSNOB or REBURP]
;spnam22 : file name for the selective 180 refocusing shaped pulse PS [RSNOB or REBURP]
;spnam32 : file name for the adiabatic shaped pulse using in first ZQF [CHIRP]
; smoothed chirp (low to high, 20% smoothing, 1000 points, 20KHz)
;spnam34 : file name for the adiabatic shaped pulse using in last ZQF [CHIRP]
; smoothed chirp (low to high, 20% smoothing, 1000 points, 20KHz)

;GRADIENT SHAPE
;gpnam1 : SMSQ10.100
;gpnam2 : SMSQ10.100
;gpnam3 : SMSQ10.100
;gpnam4 : SMSQ10.100
;gpnam8 : SMSQ10.100
;gpnam11: RECT.1

;GRADIENT STRENGTH
;gpz0 : first ZQF gradient [3%]
;gpz1 : CTP gradient [13 or 23%]
;gpz2 : homospoil gradient [37%]
;gpz3 : saturation recovery gradient [47%]
;gpz4 : saturation recovery gradient [63%]
;gpz8 : saturation recovery gradient [31%]
;gpz10: last ZQF gradient [4%]
;gpz11: gradient under SALTIRE pulse [1-3%]
;gpz20: z-encoding gradient in ZS element [0-1%]

;CONSTANTS
;cnst4 : Number of drop points [4]
;cnst12: chemical shift for first frequency of selective pulse (offset, in ppm)
;cnst13: chemical shift for second frequency of selective pulse (offset, in ppm)
;cnst22: chemical shift for selective pulse (offset, in ppm)
;cnst41: Bandwidth for selective pulse (Hz)
;cnst42: Bandwidth for ASR selective pulse (Hz)
;cnst48: beta flip angle of the PSYCHE pulse [20]
;cnst50: sweep-width of the PSYCHE pulse [10 kHz]
;cnst51: duration of the PSYCHE pulse [30 ms]

;;;; WAVEMAKER ;;;;
;sp12:wvm:mjs_multi_rsnob:f1 rsnob(cnst41 Hz, cnst12 ppm; NPOINTS=1000; PA=0.5; PHI=0;) rsnob(cnst41 Hz, cnst13 ppm; NPOINTS=1000; PA=0.5; PHI=0;)
;sp22:wvm:mjs_asr_rsnob:f1 rsnob(cnst42 Hz; NPOINTS=1000; PA=0.5; PHI=0;)
;sp32(p32):wvm:mjs_ZQS1:f1 wurst-80(20 kHz, 10 ms; NPOINTS=10000, L2H, Q=11) ss=5.0us;

```
;sp34(p34):wvm:mjs_ZQS2:f1 wurst-80(20 kHz, 30 ms; NPOINTS=10000, L2H, Q=11) ss=5.0us;
;sp50(p50):wvm:mjs_psyche:f1 cawurst-40(cnst50 kHz, cnst51 ms; L2H) cawurst-40(cnst50 kHz, cnst51 ms; H2L)
ss=2.0us

;OTHER
;ZGOPTNS : -DZS (Zangger-Sterk), -DdSALTIRE (PSYCHE on)
;td1 : number of delays in VDLIST
;vd : variable delay, taken from vd-list
;define VDLIST
;this pulse program produces a ser-file (PARMOD = 3D)
;in0 : 1/(2 * SW) = DW
;nd0 : 2
;NS: 16 * n, total number of scans: NS * TD0
;DS: 16
```

3. PUREST-T_2 - Periodic refocussing of J evolution (PROJECT) with pure shift yielded by CHIRP excitation (PSYCHE)

```
; PUREST-T2
;
; Pure Shift Relaxation Encoded Selective TOCSY (PUREST-T2)
; Using PROJECT T2 relaxation weighting and PSYCHE J refocussing element
; DIPSI-2 with ZQF
;
; University of Manchester
; Manchester NMR methodology group
; https://www.nmr.chemistry.manchester.ac.uk/
;
; Mathias Nilsson
; Mathias.Nilsson@manchester.ac.uk
;
; Marshall Smith
; marshall.smith@manchester.ac.uk

;Data can be reconstructed using a macro available at http://nmr.chemistry.manchester.ac.uk
;Avance II+/III Version
;Topspin 4.x
;
;$CLASS=HighRes
;$DIM=3D
;$TYPE=
;$SUBTYPE=
;$COMMENT=

#include <Avance.incl>
#include <Delay.incl>
#include <Grad.incl>

define delay tauA
define delay tauB

;PULSES
"p2=p1*2"
"p3=p1"
"p18=1m"
```

```
"p19=1m"
"p11=p40"

"FACTOR1=(d9/(p6*115.112))/2"
"l1=FACTOR1*2"

;DELAYS

"in0=inf1/2"
"d0=0"
"d11=30m+1s/(cnst12)-1s/(cnst12)"
"d11=30m"
"tauA=in0/2-p18-d18-50u"
"tauB=dw*2*cnst4"
"d18=1m"
"d19=1m"

"spoff12=bf1*(cnst12/1000000)-o1"
"spoff32=0"
"spoff34=0"
; Zero Quantum Filter Pulse Calculation
"p33=1000000.0/(cnst53*4)"
"cnst33= (p33/p1) * (p33/p1)"
"spw32=plw1/cnst33"
"p35=1000000.0/(cnst54*4)"
"cnst35= (p35/p1) * (p35/p1)"
"spw34=plw1/cnst35"

; SALTIRE PULSE
"cnst50=(cnst20/360)*sqrt((2*cnst21)/(p40/2000000))"
"p30=1000000.0/(cnst50*4)"
"cnst31= (p30/p1) * (p30/p1)"
"spw40=plw1/cnst31"
"spoff40=0"

;Acquistion
"acqt0=0"
baseopt_echo

1 ze
2 30m pl1:f1

  10u UNBLKGRAMP
  p8 ph21
  p8 ph22
  p15:gp8*-1
  d15 BLKGRAMP

  50u LOCKH_OFF
  d1
  50u LOCKH_ON
  50u UNBLKGRAMP

  p1 ph1

3 d20
  p2 ph2
  d20
  p3 ph3
```

```
  d20
  p2 ph2
  d20
  lo to 3 times c

4 p16:gp1
  d16 pl0:f1
  p12:sp12:f1 ph4                                   ;selective 180r
  p16:gp1
  d16 pl1:f1

5 p1 ph5
  20u gron0 pl0:f1
  (p32:sp32 ph6):f1
  10u groff
  200u pl10:f1

                                                        ;begin DIPSI2
6 p6*3.556 ph23
  p6*4.556 ph25
  p6*3.222 ph23
  p6*3.167 ph25
  p6*0.333 ph23
  p6*2.722 ph25
  p6*4.167 ph23
  p6*2.944 ph25
  p6*4.111 ph23

  p6*3.556 ph25
  p6*4.556 ph23
  p6*3.222 ph25
  p6*3.167 ph23
  p6*0.333 ph25
  p6*2.722 ph23
  p6*4.167 ph25
  p6*2.944 ph23
  p6*4.111 ph25

  p6*3.556 ph25
  p6*4.556 ph23
  p6*3.222 ph25
  p6*3.167 ph23
  p6*0.333 ph25
  p6*2.722 ph23
  p6*4.167 ph25
  p6*2.944 ph23
  p6*4.111 ph25

  p6*3.556 ph23
  p6*4.556 ph25
  p6*3.222 ph23
  p6*3.167 ph25
  p6*0.333 ph23
  p6*2.722 ph25
  p6*4.167 ph23
  p6*2.944 ph25
  p6*4.111 ph23
  lo to 6 times l1
                                                      ;end DIPSI2
```

```
 p17:gp2
 500u
 10u gron10 pl0:f1
 (p34:sp34 ph6):f1                    ;Zero Quantum Filter
 200u groff
 10u pl1:f1

7 p1 ph7
 d0

 tauA
 50u
 p18:gp3
 d18
 p2 ph8
 p18:gp3
 d18
 50u
 tauA

 tauB

 50u
 d19
 p19:gp4
 d19
 10u pl0:f1
 ( center (p40:sp40 ph9):f1 (p11:gp11) )     ;PSYCHE element
 d19
 10u pl1:f1
 p19:gp4
 d19
 50u BLKGRAMP

 d0

 ;End PSYCHE
 go=2 ph31
 30m mc #0 to 2
          F1QF(id0)
          F2QF(ivc)
exit
 50u LOCKH_OFF


ph1=0
ph2=0
ph3=1
ph4=0 1 2 3
ph5=0
ph6=0
ph7=0
ph8=0
ph9=0 0 0 0 1 1 1 1 2 2 2 2 3 3 3 3
ph21=0
ph22=1
ph23=3
ph25=1
ph31=0 2 0 2 2 0 2 0
```

```
;POWER LEVEL
;pl0  : zero power (0W)
;pl1  : power level for pulse (default)
;pl10 : power level for TOCSY-spinlock
;spw12 : power level of refocusing shaped pulse
;spw32 : power level of adiabatic pulse of first ZQF element
;spw34 : power level of adiabatic pulse of last ZQF element
;spw40: power level of Saltire pulse

;PULSE DURATION
;p1  : 90 degree high power pulse
;p2  : 180 degree high power pulse
;p6  : 90 degree low power pulse
;p8  : Saturation recovery pulse                                    [1-1.5 msec]
;pl0 : zero power [O W]
;p12 : 180 degree refocusing shaped pulse Selective TOCSY
;      choose p12 according to desired selectivity
;p32 : first ZQF 180 degree inversion shaped pulse (adiabatic)      [10 msec]
;p34 : second ZQF 180 degree inversion shaped pulse (adiabatic)     [30 msec]
;p40: f1 channel -  duration of SALTIRE pulse

;GRADIENT DURATION
;p11: duration of gradient under SALTIRE pulse
;p16 : duration of CTP gradients for Selective pulse                [1 msec]
;p17 : duration of CTP gradients for z-TOCSY                        [1 msec]
;p18 : saturation recovery gradient pulse                           [1 msec]
;p19 : duration of CTP gradients for PSYCHE selective pulse [1 msec]

;DELAY
;d0  : incremented delay
;d1  : relaxation delay;                                            [2-10 s]
;d9  : TOCSY mixing time                                            [50-200 ms]
;d11 : delay for disk I/O                                           [30 msec]
;d15 : recovery delay for homospoil gradient                        [1 ms]
;d16 : recovery delay for gradients of selective pulse CTP          [1 ms]
;d17 : selective spin-echo delay in selective-TOCSY block           [200 us]
;d18 : recovery delay for saturation gradients                      [1ms]
;d19 : recovery delay for CTP gradients for ZS selective pulse      [1 msec]
;d20 : inter pulse delay in PROJECT element                         [3-5 ms]

;PULSE SHAPE
;spnam12 : file name for the selective 180 refocusing shaped pulse selective TOCSY     [RSNOB or REBURP]
;spnam32 : file name for the adiabatic shaped pulse using in first ZQF                 [CHIRP]
;        smoothed chirp (low to high, 20% smoothing, 1000 points, 20KHz)
;spnam34 : file name for the adiabatic shaped pulse using in last ZQF                  [CHIRP]
;        smoothed chirp (low to high, 20% smoothing, 1000 points, 20KHz)
; spnam40 : file name for the adiabatic shaped pulse for PSYCHE element                [SALTIRE]
;
;GRADIENT SHAPE
;gpnam1 : SMSQ10.100
;gpnam2 : SMSQ10.100
;gpnam3 : SMSQ10.100
;gpnam4 : SMSQ10.100
;gpnam8 : SMSQ10.100
;gpnam11 : RECT.1

;GRADIENT STRENGTH
;gpz0 : first ZQF gradient                        [3%]
```

;gpz1 : CTP gradient [13 or 23%]
;gpz2 : homospoil gradient [37%]
;gpz3 : CTP gradient [47%]
;gpz4 : CTP gradient for SALTIRE pulse [67%]
;gpz8 : saturation recovery gradient [31%]
;gpz10: last ZQF gradient [4%]
;gpz11 : Gradient for PSYCHE J refocussing element [1-3%]

;CONSTANTS
;cnst4: number of drop points [4]
;cnst12: chemical shift for selective pulse (offset, in ppm)
;cnst20: desired flip angle for saltire pulse element (degree) (normally 10-50)
;cnst21: bandwidth of saltire pulse (Hz) (normally 10000 Hz)
;cnst53: GammaB1/2*pi of first adiabatic ZQF shaped pulse
;cnst54: GammaB1/2*pi of last adiabatic ZQF shaped pulse

;OTHER
;PSYCHE: set O1 on in the center of the spectral window to be excited
;td1 number of t1 increments [16-64]
;td2 : number of delays in VDLIST
;vc : variable counter, taken from vc-list
;define VDLIST
;this pulse program produces a ser-file (PARMOD = 3D)
;NS: 16 * n, total number of scans: NS * TD0
;in0 : 1/(2 * SW) = DW
;nd0 : 2
;DS: 16
